# Exploration of comorbidity mechanisms and potential therapeutic targets of rheumatoid arthritis and pigmented villonodular synovitis using machine learning and bioinformatics analysis

**DOI:** 10.3389/fgene.2022.1095058

**Published:** 2023-01-06

**Authors:** Hongquan Heng, Dazhuang Li, Wenxing Su, Xinyue Liu, Daojiang Yu, Zhengjun Bian, Jian Li

**Affiliations:** ^1^ Department of Orthopedics, The Second Affiliated Hospital of Soochow University, Suzhou, China; ^2^ Department of Orthopedics, The Affiliated Hospital of Yangzhou University, Yangzhou University, Yangzhou, China; ^3^ Department of Plastic and Burn Surgery, The Second Affiliated Hospital of Chengdu Medical College (China National Nuclear Corporation 416 Hospital), Chengdu, China; ^4^ Department of Radiology, Wangjiang Hospital of Sichuan University, Chengdu, China

**Keywords:** rheumatoid arthritis, pigmented villonodular synovitis, weighted gene co-expression network analysis, machine learning, immune cell infiltration, hub gene

## Abstract

**Background:** Rheumatoid arthritis (RA) is a chronic autoimmune disease. Pigmented villonodular synovitis (PVNS) is a tenosynovial giant cell tumor that can involve joints. The mechanisms of co-morbidity between the two diseases have not been thoroughly explored. Therefore, this study focused on investigating the functions, immunological differences, and potential therapeutic targets of common genes between RA and PVNS.

**Methods:** Through the dataset GSE3698 obtained from the Gene Expression Omnibus (GEO) database, the differentially expressed genes (DEGs) were screened by R software, and weighted gene coexpression network analysis (WGCNA) was performed to discover the modules most relevant to the clinical features. The common genes between the two diseases were identified. The molecular functions and biological processes of the common genes were analyzed. The protein-protein interaction (PPI) network was constructed using the STRING database, and the results were visualized in Cytoscape software. Two machine learning algorithms, least absolute shrinkage and selection operator (LASSO) logistic regression and random forest (RF) were utilized to identify hub genes and predict the diagnostic efficiency of hub genes as well as the correlation between immune infiltrating cells.

**Results:** We obtained a total of 107 DEGs, a module (containing 250 genes) with the highest correlation with clinical characteristics, and 36 common genes after taking the intersection. Moreover, using two machine learning algorithms, we identified three hub genes (PLIN, PPAP2A, and TYROBP) between RA and PVNS and demonstrated good diagnostic performance using ROC curve and nomogram plots. Single sample Gene Set Enrichment Analysis (ssGSEA) was used to analyze the biological functions in which three genes were mostly engaged. Finally, three hub genes showed a substantial association with 28 immune infiltrating cells.

**Conclusion:** PLIN, PPAP2A, and TYROBP may influence RA and PVNS by modulating immunity and contribute to the diagnosis and therapy of the two diseases.

## Introduction

RA is a chronic autoimmune disease that primarily affects the joints and is characterized by progressive, symmetrical inflammation of the joints, ultimately leading to destruction of articular cartilage, bone erosion, and disability (Smolen et al., 2016). The histological manifestations of RA are mainly three stages: cell proliferation, fibrin exudation, and inflammatory infiltration (Aigner and McKenna, 2002). The disease is primarily caused by the transport of hyperplastic synovial tissue fibroblasts, T and B lymphocytes, neutrophils and monocytes into the synovial tissue (Muller-Ladner et al., 2005).

PVNS, also known as tenosynovial giant cell tumor, is a rare joint disease. The annual incidence of PVNS is 1.8 per million and is increasing each year as awareness of the disease grows (Xie et al., 2015). It is characterized by inflammatory synovitis, synovial cell hyperplasia, and massive monocyte-derived osteoclast accumulation in joint synovial tissue (Rubin, 2007). It is a classic single-joint disease that frequently affects the knee, followed by the hip, ankle, shoulder, and elbow (Myers and Masi, 1980; Abdul-Karim et al., 1992; Chebib et al., 2018). The histological features of PVNS are fibrous matrix hyperplasia, macrophage infiltration, and hemosiderin deposition (Dorwart et al., 1984).

Both RA and PVNS are joint diseases, and their common feature is synovial hyperplasia due to excessive proliferation of synovial cells. Both RA and PVNS have an inflammatory environment (Berger et al., 2005). A study comparing the pathological features of RA and PVNS found hyperplasia of macrophages and fibroblasts in the lesioned synovial tissue (Berger et al., 2005). On arthroscopy and pathological examination, the villous nodular tissue exhibited more typical features of PVNS. Intra-articular injection of TNF-α inhibitors showed significant therapeutic effects in both RA and PVNS (Fiocco and Punzi, 2011). The lack of attention to PVNS has led to an increasing incidence of coexisting PVNS and RA, and the ability to correctly identify PVNS and RA will have a direct impact on patient outcome, so there is an urgent need to develop new biomarkers to identify these two diseases.

In this study, we extracted 18 RA samples and 11 PVNS samples. After normalization of the GSE3698 dataset, 107 differentially expressed genes (60 up-regulated and 47 down-regulated) were identified. In this way, the genes that are differentially expressed in RA and PVNS were analyzed, and the common target for diagnosing RA and PVNS was developed.

## Materials and methods

### Data collection and standardization

The GSE3698 (Finis et al., 2006) dataset was acquired from the GEO database (http://www.ncbi.nlm.nih.gov/geo) (Edgar et al., 2002), the dataset was based on the GPL3050 (Human Unigene3.1 cDNA Array 37.5K v1.0) and 18 RA samples and 11 PVNS samples were extracted from this dataset. We used R (version 4.2.0) to process the data and explore the downstream functional expressions. Using the “limma” package (Ritchie et al., 2015), the expression matrix was constructed, and then the dataset was normalized by taking log2 and utilizing the normalize Between Arrays function.

### Identification of DEGs

First, the dataset of GSE3698 was transformed into an expression matrix using the “limma” packages, and then differentially expressed genes (DEGs) were identified using the screening criteria adjust *p*-value <.05 and abs (logFC) > 0.5, Second, the “pheatmap” package was used to display the 30 most variable genes among PVNS and RA samples. Lastly, the “ggplot2” package (Wickham, 2009) was employed to generate a volcanic map depicting which genes were turned up or down.

### WGCNA screening for key module genes

Gene association patterns between diverse samples can be characterized using the systems biology method known as WGCNA (Langfelder and Horvath, 2008). To weed out samples that might be inappropriate, we used a mean FPKM = 0.5 filtering criterion. Then, with the scale-free network concept, the weighting coefficient was determined. In order to calculate the dynamic tree cutting procedure, set the red line to 0.9, the module cut height to 0.25, and the minimum module gene count to 40. At last, for major modules related to clinical characteristics, module membership (MM) and gene significance (GS) were computed.

### Functional enrichment analysis of common genes

The DEGs and selected key module genes were intersected using the “randomcoloR” and “venn” packages to identify common genes. To explore the biological functions of the common genes of PVNS and RA, we analyzed the Gene Ontology (GO) and Kyoto Encyclopedia of Genes and Genomes (KEGG) pathways *via* “ClusterProfiler”, “ggnewscale” and “DOSE” packages (Yu et al., 2012; Yu et al., 2015; Campitelli, 2020). Adjusted *p*-value <.05 was considered significant.

### PPI network construction and analysis of common genes

Search Tool for the Retrieval of Interacting Genes (STRING; http://string-db.org) (version 11.5) (Franceschini et al., 2013) could be used to search for interactions between proteins of interest with the goal of creating PPI networks with complicated relationships. Interactions with a combined score greater than 0.40 were statistically significant. This PPI network was represented using Cytoscape (http://www.cytoscape.org) (version 3.9.1) (Smoot et al., 2011). The core common genes were found by using the CytoNCA (Tang et al., 2015) plug-in in Cytoscape. Here, we applied betweenness (BC) to determine core common genes. Subsequently, we generated a network diagram of the core common genes with the closest associations.

### Hub genes identification with machine learning

Machine learning methods can improve the accuracy of gene screening. On one hand, LASSO logistic regression algorithm (Tibshirani, 1996) put 36 common genes in the common multiple regression, increased the penalty function, and continuously compressed the coefficients, thus streamlining the model and filtering out the number of genes with the best fit. On the other hand, using the “glmnet” package (Friedman et al., 2010), LASSO regression was used to discover hub genes. What’s more, the RF algorithm (Breiman, 2001) was conducted to screen hub genes by using the “randomForest” package (Liaw and Wiener, 2002). Finally, overlapping genes among 36 common genes generated *via* LASSO regression and RF algorithm were considered as hub genes in PVNS and RA. The GeneCards database (http://www.genecards.org/) was used to find relevant genes, proteins, and disease connections.

### Evaluation of the diagnostic efficacy of hub genes

Combined with the screened hub genes, logistics regression was used to construct nomogram, Similarly, the hub genes were tested to see whether they might be used to distinguish PVNS samples from control samples using the “pROC” R program (Robin et al., 2011).

### Biological process and immune infiltration analysis of hub genes

To begin with, the “ggplot2”, “limma”, and “pheatmap” packages were used to investigate the functional pathways enriched with hub genes. ssGSEA is a method for investigating the absolute enrichment of hub genes in a dataset. Furthermore, ssGSEA was performed using the “GSEABase” package (Merico et al., 2010) and “GSVA” package (Hänzelmann et al., 2013) to investigate differences in immune cells’ expression between PVNS and RA samples and immune infiltration of hub genes.

### Evaluation of expression differences of hub genes

The “PerformanceAnalytics” (Peterson et al., 2018) and “circlize” (Gu et al., 2014) packages were used to explore the correlation analysis on hub genes, and the “corrplot” package was used to visualize the results. Moreover, to investigate differences in hub genes, statistical validation of differential expression analysis was carried out using the “limma” package.

## Results

### Identification of DEGs

**FIGURE 1:**
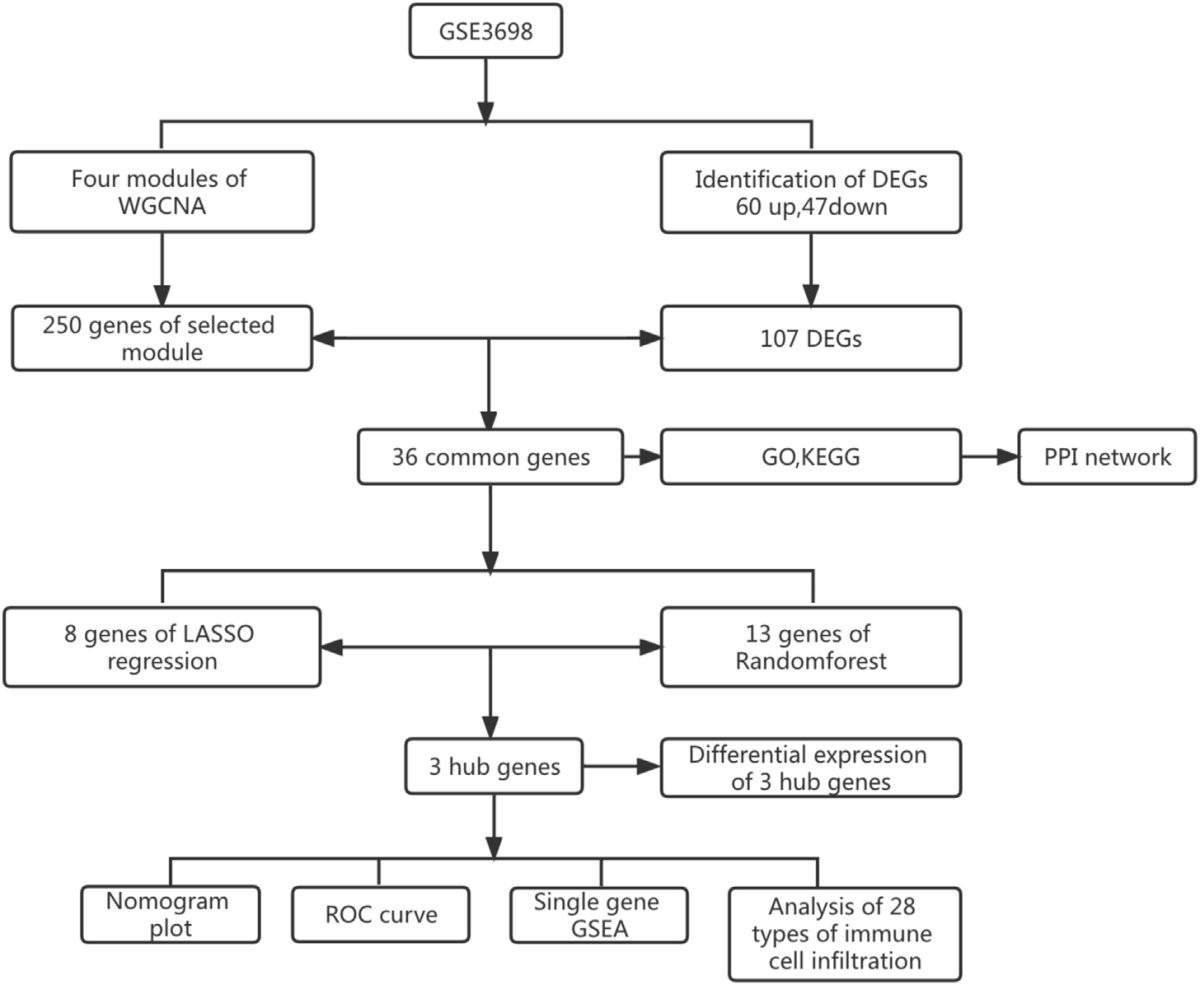
Research process flow diagram.

**FIGURE 2:**
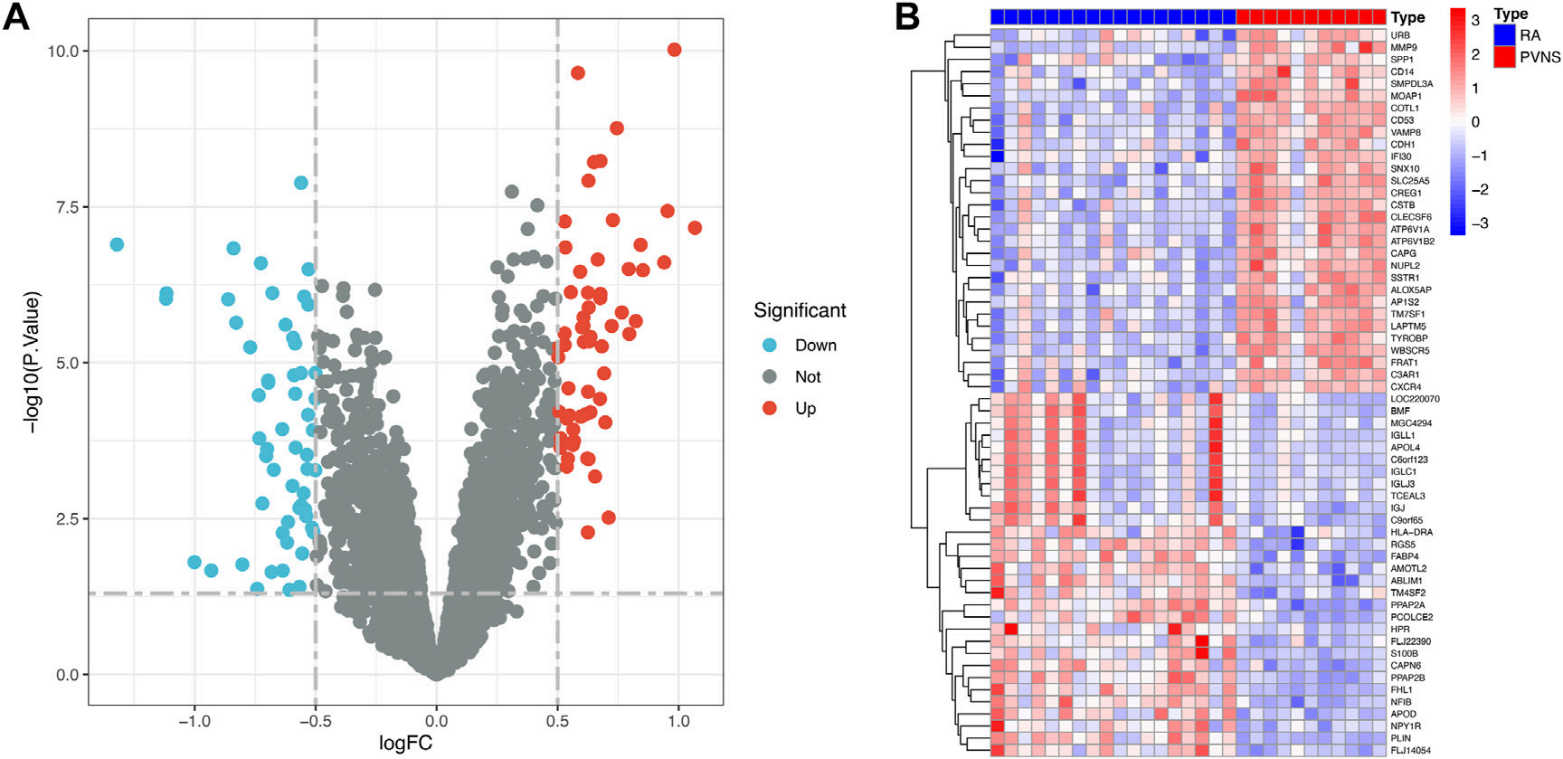
**(A)** The volcano map of GSE3698. **(B)** The heatmap of GSE3698.

The research process is shown in Figure 1. After normalizing the GSE3698 dataset (The normalized boxplots of the dataset were shown in Supplementary Figure S1), 107 differentially expressed genes were identified, consisting of 60 up-regulated and 47 down-regulated genes. The statistics were shown on a map of volcanoes (Figure 2A). Figure 2B displayed a cluster heatmap based on the 30 most differentially expressed genes.

### Acquisition of key modules and common genes

**FIGURE 3:**
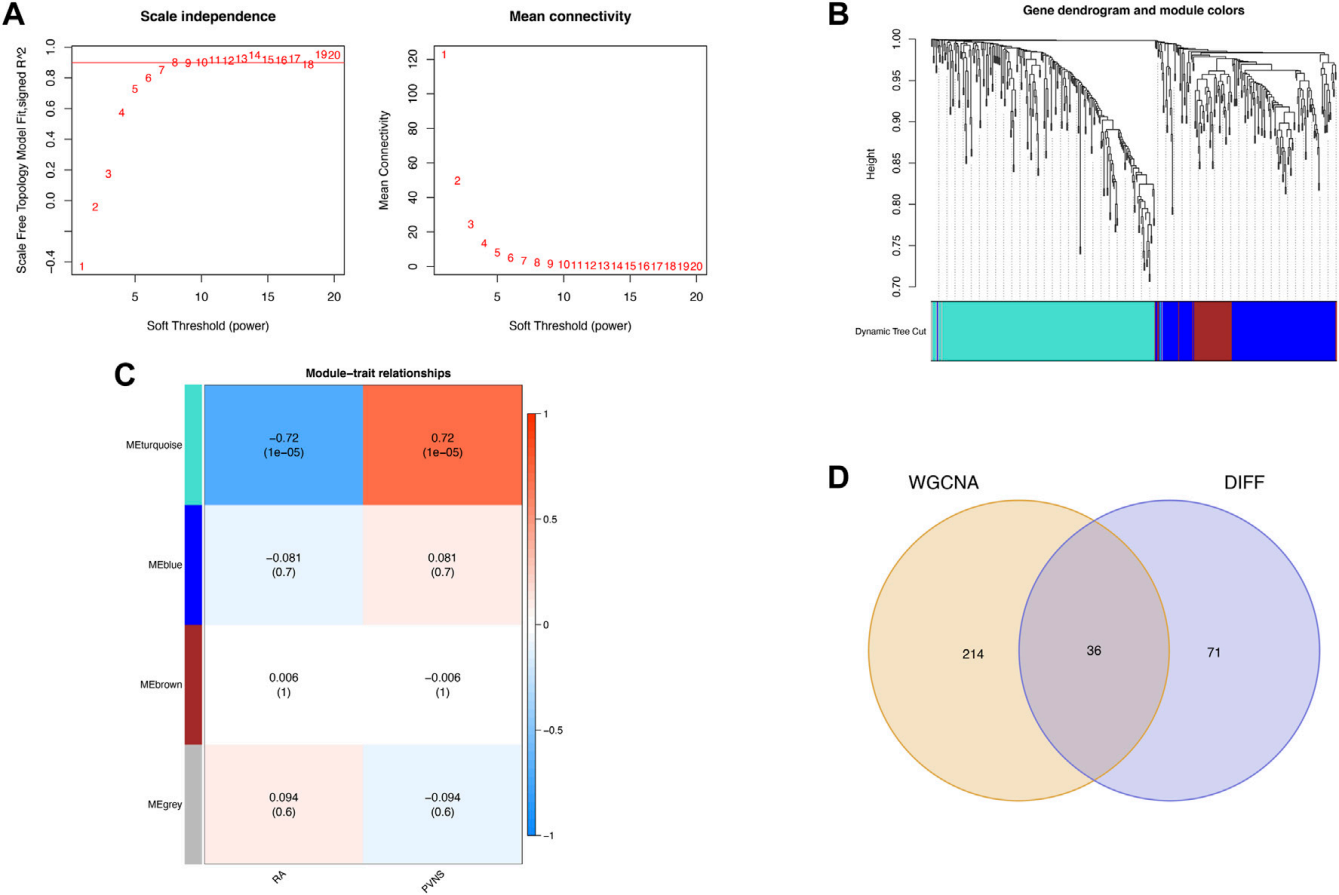
**(A)** WGCNA provides the definition for soft threshold power. For different soft threshold powers (β), scale-free indices and mean connectedness are examined **(B)** The method of hierarchical clustering is used to find gene co-expression clusters. Each branch of the tree diagram represented a gene, and genes that belong to the same module have the same coloring. **(C)** Four modules with different colors are obtained by linking the clinical characteristics of PVNS and RA, combining modules with a feature factor greater than 0.45 and setting the minimum number of module genes to 40 for identification. **(D)** Venn diagram demonstrates the intersection of common genes obtained by WGCNA and DEGs.

WGCNA was used to search for key module genes to identify those having the highest correlation to clinical characteristics. The appropriate soft threshold was determined to be 8 (Figure 3A. To satisfy the scale-free network topology, we select a soft threshold power of 8 with *R* ^2^ = 0.89, as demonstrated in Supplementary Figure S2). Then, the dynamic tree cutting technique obtained a total of 4 key modules by setting MEDissThres to 0.25 and minModuleSize to 45 (Figure 3B). Furthermore, analysis of correlation revealed that the MEturquoise module was the most significant module for PVNS (Figure 3C). Finally, the 107 DEGs were intersected with the 250 genes acquired by WGCNA analysis to produce 36 common genes of PVNS and RA (Figure 3D).

### Functional enrichment analysis and PPI networks

**FIGURE 4:**
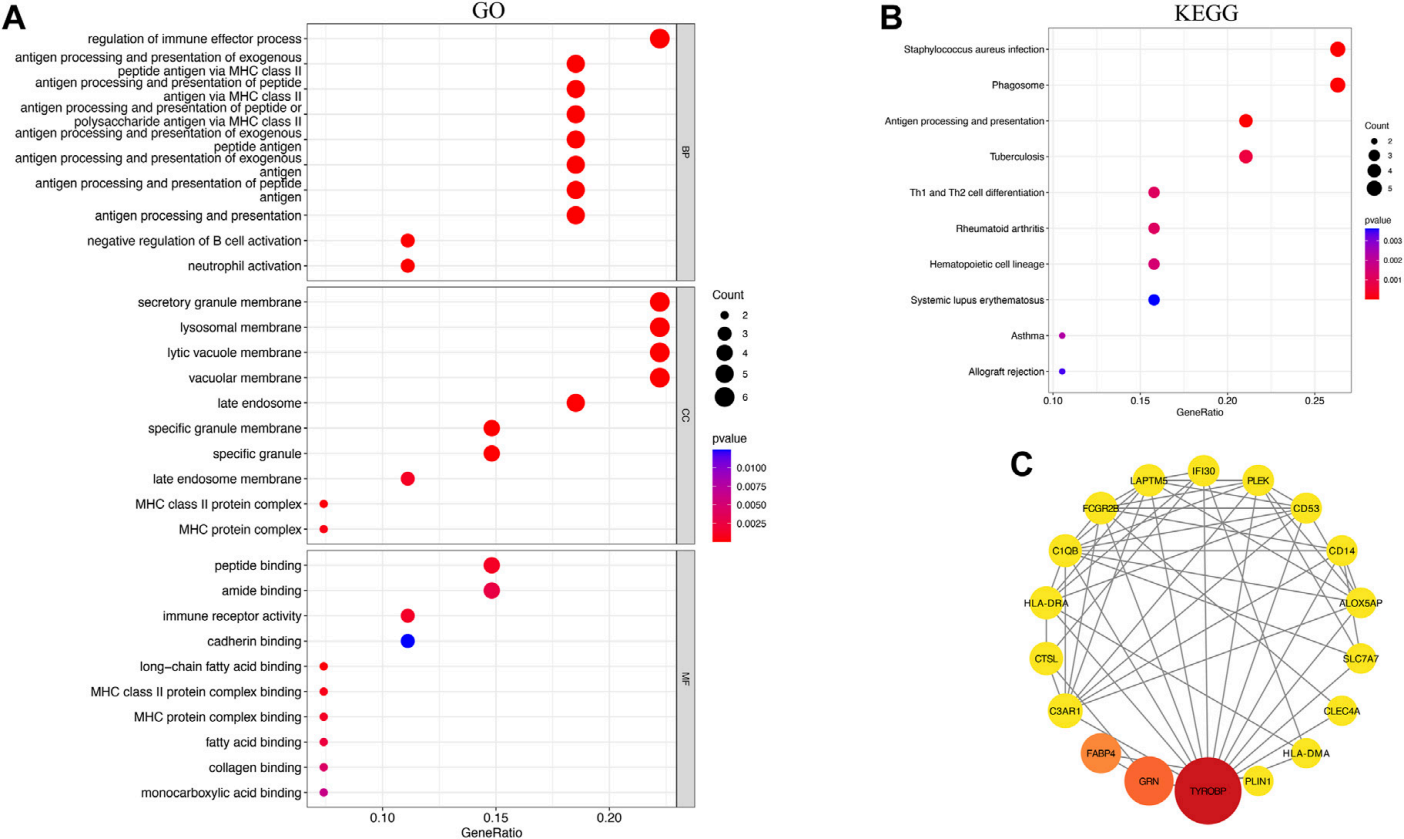
**(A)** Results of GO analysis of the top 10 common genes, including BP, MF and CC **(B)** Analysis of KEEG enrichment revealed signaling pathways strongly related with PVNS and RA. **(C)** PPI network constructed using the STRING database and Cytoscape. The wider the circle, the greater its significance, and the redder the color, the greater its significance.

To investigate possible shared biological pathways and mechanisms between PVNS and RA, we performed Gene Ontology (GO) and Kyoto Encyclopedia of Genes and Genomes (KEGG) pathway enrichment of 36 common genes. GO analysis was notably enriched in regulation of immune effector process, antigen processing and presentation of exogenous peptide antigen *via* MHC class II, antigen processing and presentation of peptide or polysaccharide antigen *via* MHC class II, secretory granule membrane, lysosomal membrane, immune receptor activity and peptide binding (Figure 4A). The KEGG enrichment analysis showed that antigen processing and presentation, *staphylococcus aureus* infection, phagosome, and Th1 and Th2 cell differentiation may play a significant role in PVNS and RA (Figure 4B). To examine the interrelationships of common genes between PVNS and RA, we imported 36 common genes to the STRING database and derived interaction connections for genes with interaction score >0.4 and PPI enrichment *p*-value <.05. For visualization purposes, Cytoscape software was employed. After identifying a network of 34 nodes connected by 56 edges using the STRING database, we used the CytoNCA module in Cytoscape to calculate the degree of each gene, and then we reduced the network to 18 core nodes (Figure 4C).

### Identification of hub genes based on machine learning algorithms

**FIGURE 5:**
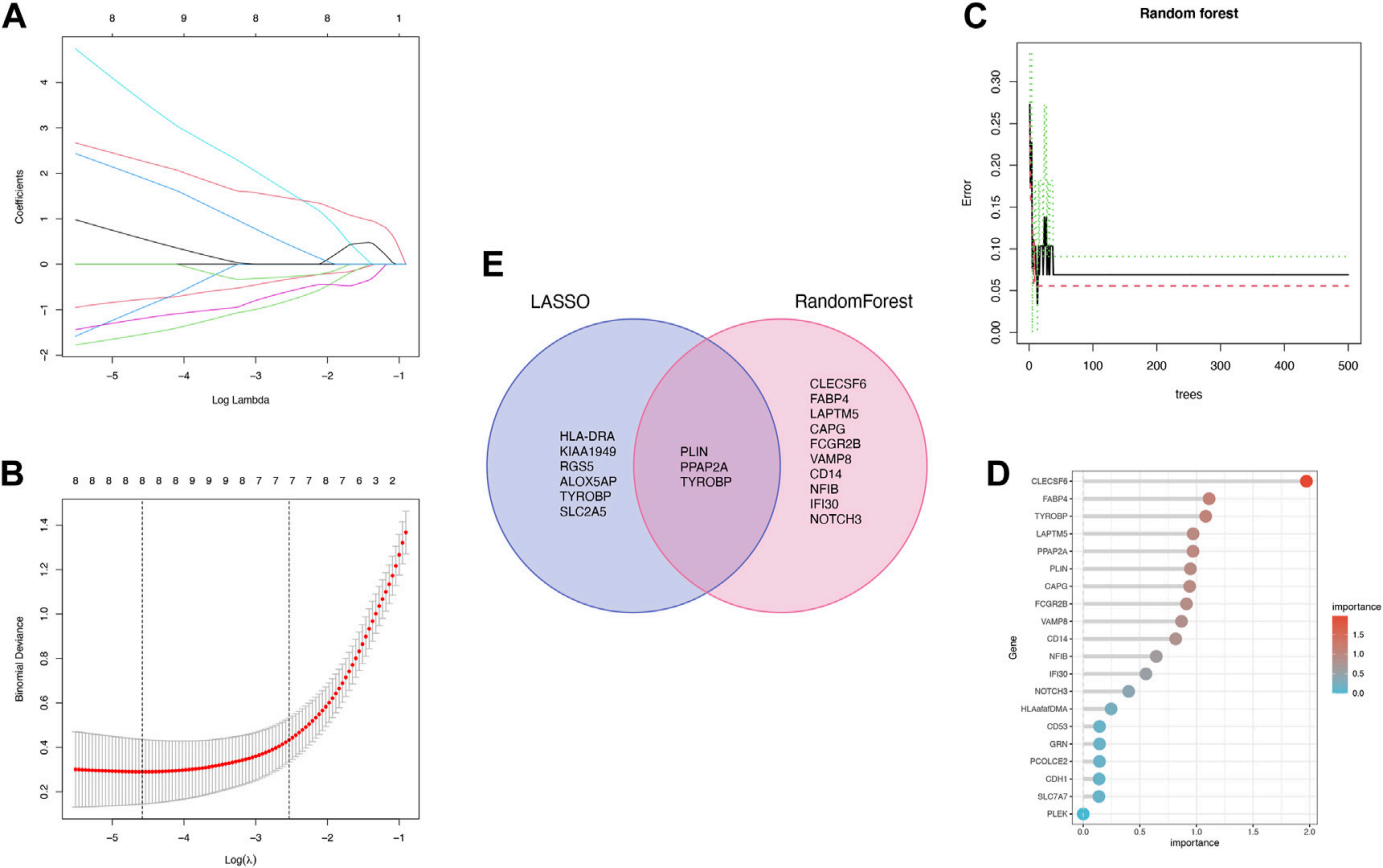
**(A,B)** LASSO logistic regression algorithm is used to retain the most predictive features and tuning parameter selection in the LASSO model **(C,D)** Identification of the relative importance *via* PVNS and RA by calculating RF. **(E)** Intersection of two machine learning genes to obtain three machine learning.

The 36 common genes were employed in the LASSO and RF analyses to screen hub genes. Firstly, The LASSO regression algorithm identified 8 out of the 36 key genes, including PLIN, PPAP2A, HLA-DRA, KIAA 1949, RGS5, ALOX5AP, TYROBP and SLC2A5 (Figures 5A, B). CLECSF6, FABP4, TYROBP, LAPTM5, PPAP2A, PLIN, CAPG, FCGR2B, VAMP8, CD14, NFIB, IFI30 and NOTCH3 were determined as the 13 most relevant variables using RF (Figures 5C, D). By overlapping the genes chosen by LASSO and RF, PLIN, PPAP2A, and TYROBP were identified as hub genes in PVNS and RA (Figure 5F). Table 1 provided their full names and functions, as found in the Gene Cards database.

**TABLE 1:** The details of hub genes in PVNS and RA.

Gene symbol	Aliases	Full name	Function
PLIN	PLIN1	Perilipin 1	Modulator of adipocyte lipid metabolism. Coats lipid storage droplets to protect them from breakdown by hormone-sensitive lipase (HSL). Unilocular lipid droplet formation by activating CIDEC. May modulate lipolysis and triglyceride levels
	FPLD4		
	PERI		
PPAP2A	PLPP1	Phospholipid Phosphatase 1	Magnesium-independent phospholipid phosphatase of the plasma membrane that catalyzes the dephosphorylation of a variety of glycerolipid and sphingolipid phosphate esters
	PAP-2a		
	LPP1		
TYROBP	DAP12	Transmembrane Immune Signaling Adaptor TYROBP	Adapter protein which non-covalently associates with activating receptors found on the surface of a variety of immune cells to mediate signaling and cell activation following ligand binding by the receptors
	KARAP		

### Verification of the diagnostic performance of hub genes

**FIGURE 6:**
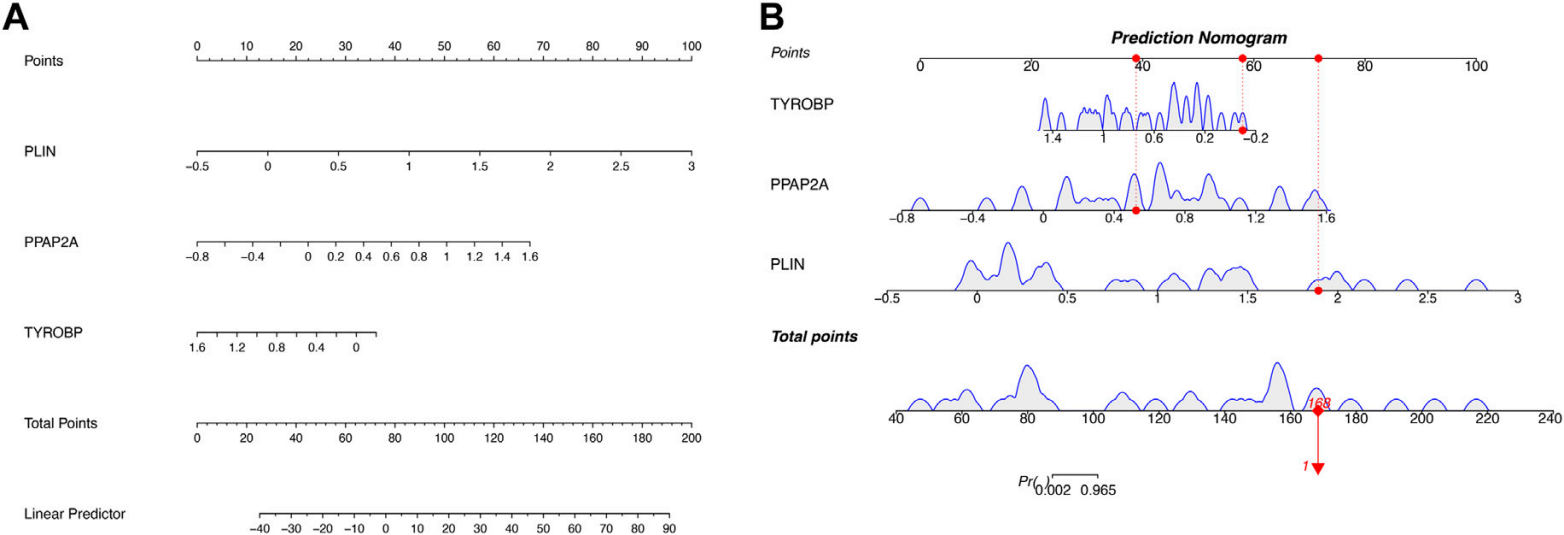
**(A)** A developed nomogram for the prognostic prediction of PVNS and RA hub genes. **(B)** This graph shows the predicted scores after aggregation of three hub genes’ proportions.

**FIGURE 7:**
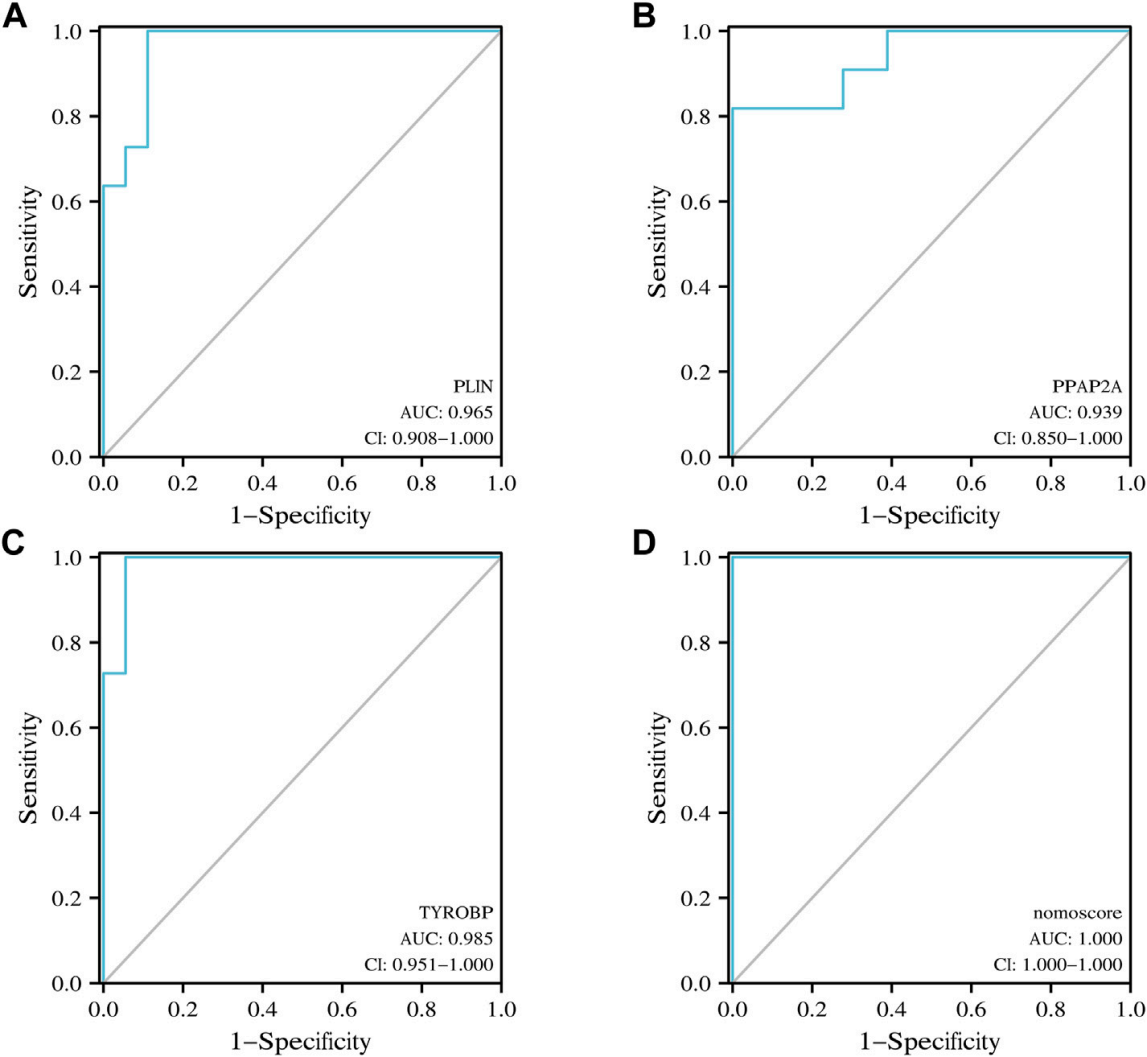
**(A–D)** ROC curve of PLIN, PPAP2A, TYROBP and nomoscore in PVNS and RA samples.

Nomogram was utilized to estimate the diagnostic implications of three hub genes, and the model comprising PLIN, PPAP2A, and TYROBP was the outcome (Figure 6A). Then, visualizing three hub genes for PVNS-related RA diagnosis using logistic regression (Figure 6B). The diagnostic utility of the hub genes was then assessed using ROC curves. The AUC values for PLIN, PPAP2A, TYROBP, and nomoscore were diagnostically effective (Figures 7A–D).

### Enrichment analysis of hub genes in PVNS and RA

**FIGURE 8:**
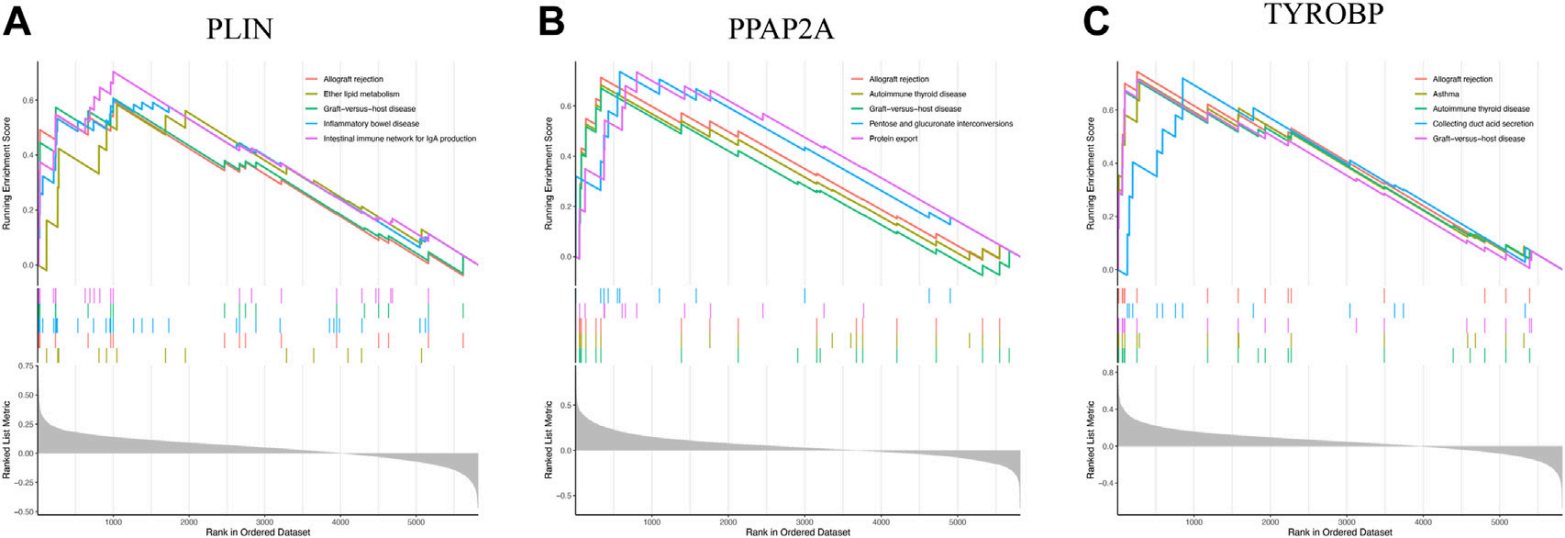
**(A)** UpGSEA results of PLIN **(B)** UpGSEA results of PPAP2A. **(C)** UpGSEA results of TYROBP.

To delve into the pathways involved in hub genes, we performed single gene GSEA analysis with the following results: PLIN mainly affected allograft rejection, ether lipid metabolism, and intestinal immune network for IgA production (Figure 8A). PPAP2A strongly influenced protein export, pentose and glucuronate interconversions, and allograft rejection (Figure 8B). TYROBP heavily impacted allograft rejection, collecting duct acid secretion, and graft−*versus*−host disease (Figure 8C).

### Immune infiltration analysis

**FIGURE 9:**
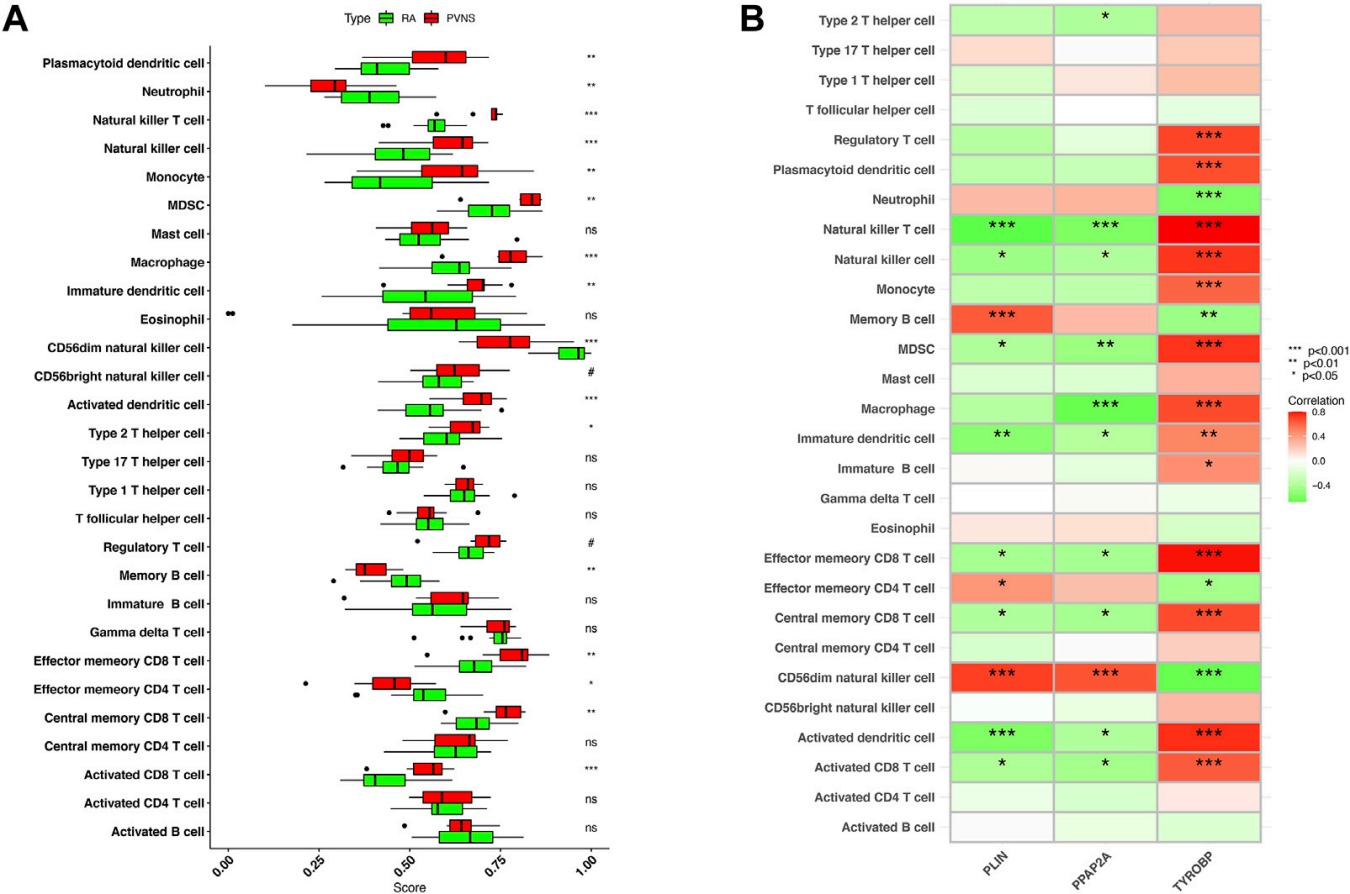
**(A)** Expression differences of 28 immune infiltrating cells in samples of PVNS and RA **(B)** Correlation between hub genes and infiltrating immune cells. Low *p*-values are green, whereas high ones are red. (nsP < 1, #*p* < 0.2, *p < 0.05, **p < 0.01, ***p < 0.001).

The correlation between hub genes and 28 kinds of immune infiltrating cells was analyzed. Initially, the expression differences of 28 immune infiltrating cells in the GSE3698 dataset were evaluated. Natural killer T cell, Natural killer cell, Macrophage, Activated dendritic cell, and Activated CD8 T cell were significantly positive correlation with PVNS (*p* < .001). PVNS was strongly connected with Plasmacytoid dendritic cell, Monocyte, MDSC, Immature dendritic cell, Effector memory CD8 T cell, and Central memory CD8 T cell (*p* < .01). Type 2 T helper cell was positively associated with PVNS (*p* < .05). On the contrary, CD56dim natural killer cell, Neutrophil and Memory B cell, Effector memory CD4 T cell were significant correlation with RA (*p* < .05) (Figure 9A). Afterwards, we looked at how 28 immune infiltrating cells were connected to 3 hub genes. CD56dim natural killer cell and Memory B cell showed a robust positive correlation with PLIN (*p* < .001). CD56dim natural killer cell had a strong correlation with PPAP2A (*p* < .001). Regulatory T cell, Plasmacytoid dendritic cell, Natural killer cell, MDSC, Macrophage, Effector memory CD8 T cell, Central memory CD8 T cell, and Activated dendritic cell were a crucial correlated with TYROP. Natural killer T cell and Activated dendritic cell were negatively correlated with PLIN (*p* < .001). Natural killer T cell and Macrophage were negatively associated with PPAP2A (*p* < .001). TYROBP and Neutrophil, CD56dim natural killer cell, had a passive correction (*p* < .001) (Figure 9B).

### Differential expression of hub genes in PVNS and RA

**FIGURE 10:**
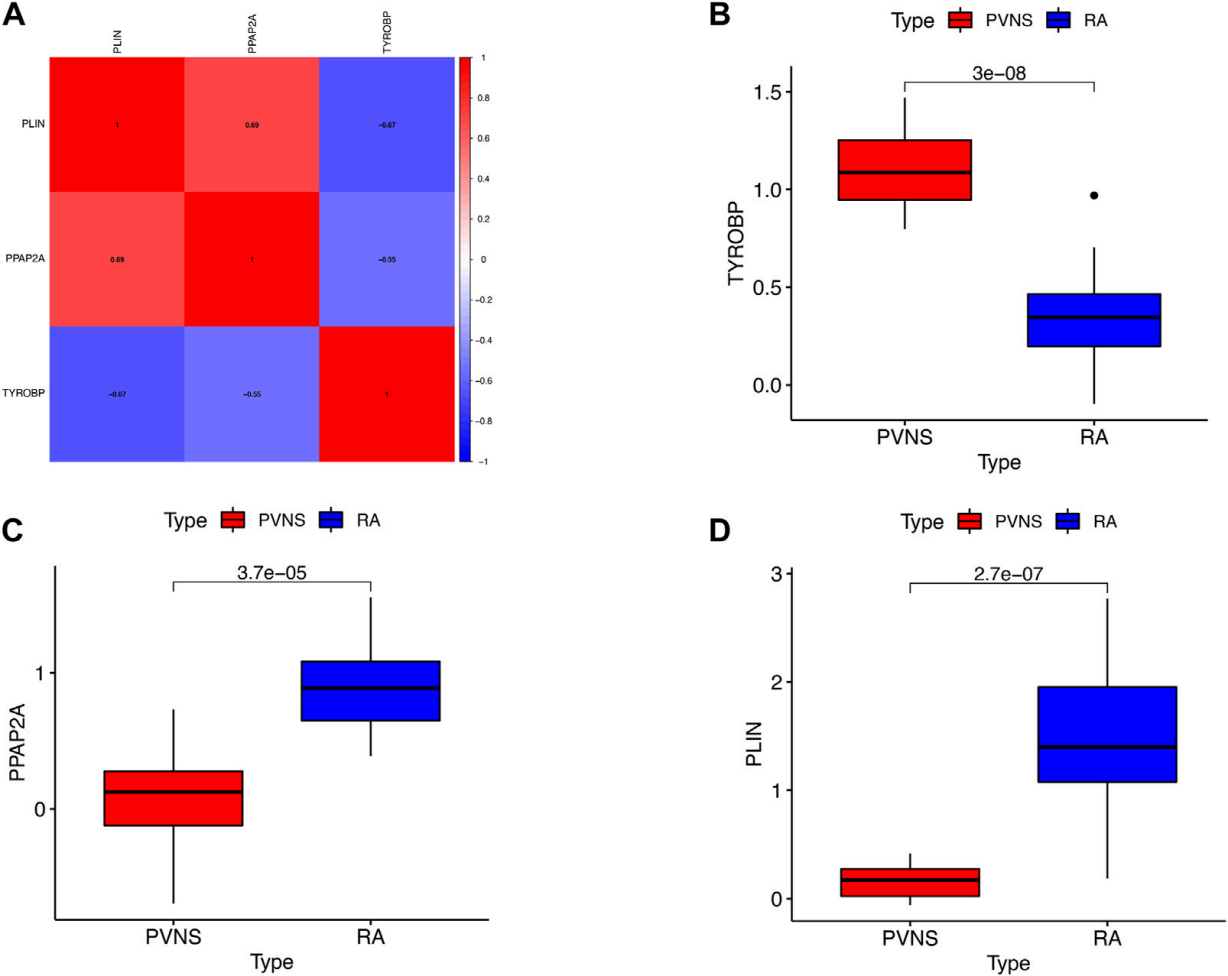
**(A)** Correlation of the three hub genes **(B)** The expression levels of upgrade hub gene in PVNS. **(C,D)** The expression levels of upgrade hub genes in RA.

To begin with, the heatmap was intended to reveal the interdependencies between hub genes, PLIN and PPAP2A had a positive correlation. PLIN was negatively associated with TYROBP (Figure 10A), the expression levels of TYROBP were obviously higher in PVNS samples than in RA samples (Figure 10B), while those of PPAP2A and PLIN were significantly lower in PVNS samples than in RA samples (Figures 10C, D). In a word, the findings demonstrated that the hub genes we examined are useful in the diagnosis of PVNS and RA.

## Discussion

Innate immune system cells, such as monocytes, macrophages, and dendritic cells (DCs), play an important role in the occurrence and development of RA disease through their functions of phagocytosis, antigen presentation, and cytokine production (McInnes et al., 2016; Narasimhan et al., 2019; Saferding and Bluml, 2020), eventually leading to the destruction of bone and cartilage (Elshabrawy et al., 2015). In particular, macrophages play a central role in the initiation and drive of RA (Udalova et al., 2016; Ardura et al., 2019; Siouti and Andreakos, 2019). The number of synovial tissue macrophages is clinically the most reliable indicator for assessing the severity of RA and response to treatment (Tak and Bresnihan, 2000; Van Raemdonck et al., 2020). In inflamed RA synovial tissue, the majority of antigen-presenting cells (APCs) are fully differentiated dendritic cells (Iwasaki and Medzhitov, 2015; Yu and Langridge, 2017), and a decrease in the number of circulating DC cells in RA patients is associated with increased inflammation (Eisenbarth, 2019). Furthermore, in RA pathology, ROS production by neutrophils at sites of inflammation leads to endothelial dysfunction and tissue damage (Cedergren et al., 2007; Cecchi et al., 2018). In the inflamed RA synovium, NK cells aggregate and lead to bone destruction (Dalbeth et al., 2004). Other studies have shown that the number of granzyme-positive NK cells is increased in early RA synovial fluid compared with osteoarthritis (Tak et al., 1994). High serum granzyme levels have been shown to be an independent predictor of early erosion in RF-positive individuals (Goldbach-Mansky et al., 2005). Under the stimulation of APC, naive CD4^+^ T cells can differentiate into different types of cells, which in turn triggers the overactivation of autoantigen T cells and B cells, which eventually leads to persistent synovitis and joint destruction (Derksen et al., 2017; Rao et al., 2017; Sparks, 2019; Lee et al., 2020; Wu et al., 2020). Th17 cells are able to produce various pro-inflammatory cytokines to promote synovitis, while Treg cells suppress inflammation and maintain immune tolerance (Bilate and Lafaille, 2012; Noack and Miossec, 2014; Shi and Chi, 2019).

PVNS may be caused by the disturbance of the CSF-1 gene at 1p13 and the COL6A3 gene at 2q35 (West et al., 2006; Cupp et al., 2007). Studies have shown that IL-1β, IL-6, TNF-α and MMP-9 are highly expressed in PVNS tissues. TNF-α stimulates the production of MMPs, which can lead to cartilage and bone destruction in PVNS (O’Keefe et al., 1998). In PVNS, the presence of macrophage, histiocyte, and plasma cell infiltration stimulates an inflammatory response (Bhatnagar et al., 2017).

The differential expression of 28 immune infiltrating cells in the GSE3698 dataset was assessed by immune infiltration analysis. We found that macrophages, plasma cells, dendritic cells, monocytes, etc. were positively correlated with PVNS. Natural killer cells, neutrophils, macrophages, etc. were significantly associated with RA. Both RA and PVNS are associated with immune infiltration, but they are also influenced by other metabolic pathways. In damaged joint tissue, MAPKs not only govern the synthesis of pro-inflammatory cytokines but also play a crucial role in the signaling cascade downstream of interleukin (IL)-1, IL-17, and tumor necrosis factor (TNF)-α receptors (McGeachy et al., 2019). PI3K/AKT interacts with the mammalian target of rapamycin (mTOR) protein, inhibits fibroblastic synoviocyte (FLS) autophagy, promotes sustained synoviocyte growth, and aggravates RA (Miryala et al., 2019). O Osteoclasts migrate, damage bones and articular cartilage *via* the PI3K/AKT signaling pathway, and eventually cause joint abnormalities and exacerbate the progression of RA) (Xin et al., 2020). The absence of Cadherin-11 inhibited PVNS and FLS migration and invasion. Moreover, the expression of cadherin-11 was upregulated by inflammatory stimuli, which in turn activated the NF-κB and MAPK signaling pathways and facilitated cartilage destruction. Cadherin-11 inhibition prevented IL-1β- and TNF-α-induced activation of the aforementioned pathways, migration and invasion of PVNS FLS, and chondrocyte injury (Cao et al., 2020).

Studies have shown that the cytological features of RA are very similar to the proliferating mononuclear synoviocytes in PVNS, and synovial cell proliferation appears to be a common feature in the pathogenesis of RA and PVNS (O’Keefe et al., 1998; Sarkissian and Lafyatis, 1999; Nanki et al., 2001), proliferating synovial cells can stimulate the expression of the macrophage marker CD68 (Aigner et al., 1998; Sarkissian and Lafyatis, 1999). Comparing the immunophenotype of proliferating synovial cells in RA and PVNS found that the same cell population was involved in the proliferative process. In localized and diffuse PVNS, macrophage-like and fibroblast-like cells proliferated, while cells expressing markers of macrophage and fibroblast-like cells hyperproliferated. In localized PVNS, a significant increase in the number of fibroblast-like synovial cells was found compared with diffuse PVNS (Flandry et al., 1994; Kobayashi et al., 1994). M1 and M2 macrophages also play a role in PVNS and RA, the detection of macrophage marker (CD68/CD163) expression showed that macrophage-positive synoviocytes were found in both RA and PVNS, In RA, CD68/CD163^+^ synoviocytes were most often found in the synovial lining layer, but in PVNS, they were more spread out (Sehgal et al., 2021). CD14^+^ cells from RA synovial fluid express low levels of M2 anti-inflammatory markers, accordingly with a high-level production of pro-inflammatory genes (Sierra-Filardi et al., 2014). Non-classical Ly6C monocytes undergo polarization into inflammatory macrophages (M1), increase disease pathogenesis, and exhibit plasticity during the resolution phase. What’s more, these cells differentiate into anti-inflammatory M2 macrophages that address the combustion environment (Misharin et al., 2014). Another study revealed that Notch signaling has a strong relationship with M1 macrophage polarization, and that inhibiting Notch signaling lowers joint tissue inflammation by inducing a switch from M1 to M2 macrophages (Sun et al., 2017). Likewise, two M2 markers remain high and stable during RA disease (Arg1 and Ym1) and M1 markers were strongly upregulated (IL-1, IL-6, and CD86) (Hofkens et al., 2013). In PVNS, a major component of the cells is composed of bystander macrophages responding to CSF1, which stimulates increased numbers of macrophages through CSF1 and also promotes monocyte infiltration, damage cell clearance, and repair (Sehgal et al., 2021).

When building a generalized linear model, the lasso machine learning algorithm can include one dimensional continuous dependent variable, multidimensional continuous dependent variables, non-negative count dependent variables, binary discrete dependent variables, and multivariate discrete dependent variables. Lasso can handle both continuous and discrete dependent variables, and in general, the data requirement (quantity) of lasso is extremely low, so the application degree is wide. This solves the problem of screening for accurate results with a small sample. Random forest is not sensitive to multivariate common linearity, and the results are relatively robust for missing data and non-equilibrium data. It can well predict the effects of up to thousands of explanatory variables (Breiman, 2001) and is known as one of the best algorithms at present (Iverson et al., 2008). This solves the problem of more robust and better prediction of data results under the same algorithm. Hence, we screened hub genes among 36 common genes using LASSO and RF analysis. By overlapping the genes selected by LASSO and RF, PLIN, PPAP2A and TYROBP were identified as central genes in PVNS and RA.

Our study found that PLIN mainly affects the intestinal immune network of allograft rejection, ether lipid metabolism, and IgA production. Existing studies have also found that PLIN1 is up-regulated in steatohepatitis caused by non-alcoholic fatty liver disease (NAFLD), but PLIN1 protein is generally not expressed in normal hepatocytes (Straub et al., 2008; Fujii et al., 2009). PLIN2 is overexpressed in patients with alcoholic steatohepatitis (Mak et al., 2008; Straub et al., 2008; Carr et al., 2014). PLIN3 upregulation has been observed in human steatotic livers (Straub et al., 2008; Pawella et al., 2014). Hypoxia-inducible protein 2 (HIG2), a target of hypoxia-inducible factor 1 (HIF1), co-localizes with PLIN2 and PLIN3 and may be a marker of hepatic hypoxia (Gimm et al., 2010). However, relatively few studies have been conducted on PLIN4 or PLIN5 in human liver, and PLIN5 may play a role in lipolysis and oxidative disposal of stored lipids (Wang et al., 2011).

The most functionally important member of the PPAP family is PPAP2A, which reduces LPA activity by dephosphorylation (Blackburn and Mansell, 2012). High levels of LPA can be detected around larger microvessels expressing autotaxin (ATX). In osteoblasts remote from microvessels, ATX was least expressed and LPA was lowest due to high PPAP2A activity (Yanai et al., 2000). Interestingly, our study also found that PPAP2A strongly affects protein export, pentose and glucuronic acid interconversion, and allograft rejection.

TYROBP is a gene located on chromosome 19. TYROBP affects allograft rejection and graft-versus-host disease by mediating cytotoxicity of natural killer cells, activation of immune cells (T cells, B cells, and macrophages) (Lanier et al., 1998; Ono et al., 2018; Zheng et al., 2020). In addition, it was found that the low expression of TYROBP can participate in the regulation of OS immune environment by participating in the activation of macrophages (Gomez-Brouchet et al., 2017; Withers et al., 2019; Wolf-Dennen et al., 2020).

At the same time, we also found a positive correlation between PLIN and PPAP2A. PLIN was negatively correlated with TYROBP, the expression level of TYROBP in PVNS samples was significantly higher than that in RA samples, while the expression levels of PPAP2A and PLIN in PVNS samples were significantly lower than those in RA samples.

## Conclusion

In a word, our findings suggest that PLIN, PPAP2A and TYROBP are associated with the occurrence and development of PVNS and RA. They are expected to become new targets and research directions for the diagnosis and treatment of PVNS and RA, thus providing new opportunities and references for improving the diagnosis and treatment level and clinical prognosis of PVNS and RA patients in the future.

## Data Availability

The original contributions presented in the study are included in the article/Supplementary Material, further inquiries can be directed to the corresponding authors.

## Supplementary material

Supplementary Figure S1 Normalized data boxplot. Different hues denote different samples. Samples are represented by rows, gene expression levels are represented by columns.

Supplementary Figure S2 Left panel shows network connectivity histogram. The right panel depicts a log-log histogram, select soft threshold power of eight and R2 greater than 0.85.

## Abbreviations

RA, Rheumatoid arthritis; PVNS, Pigmented villonodular synovitis; GEO, Gene Expression Omnibus; DEGs, Differentially expressed genes; WGCNA, Weighted gene coexpression network analysis; PPI, Protein-protein interaction; LASSO, Least absolute shrinkage and selection operator; RF, Random forest; ssGSEA, Single sample Gene Set Enrichment Analysis; MM, Module membership; GS, Gene significance; GO, Gene Ontology; KEGG, Kyoto encyclopedia of genes and genomes; DCs, Dendritic cells; APCs, Antigen-presenting cells; NAFLD, Non-alcoholic fatty liver disease; ATX, Autotaxin; HIG2, Hypoxia-inducible protein 2; HIF1, Hypoxia-inducible factor 1.
