# Supplementary figures and images for: Exploration of comorbidity mechanisms and potential therapeutic targets of rheumatoid arthritis and pigmented villonodular synovitis using machine learning and bioinformatics analysis
Source: Front Genet. 2023 Jan 6;13:1095058. doi: 10.3389/fgene.2022.1095058 (PMC9853060; doi:10.3389/fgene.2022.1095058)

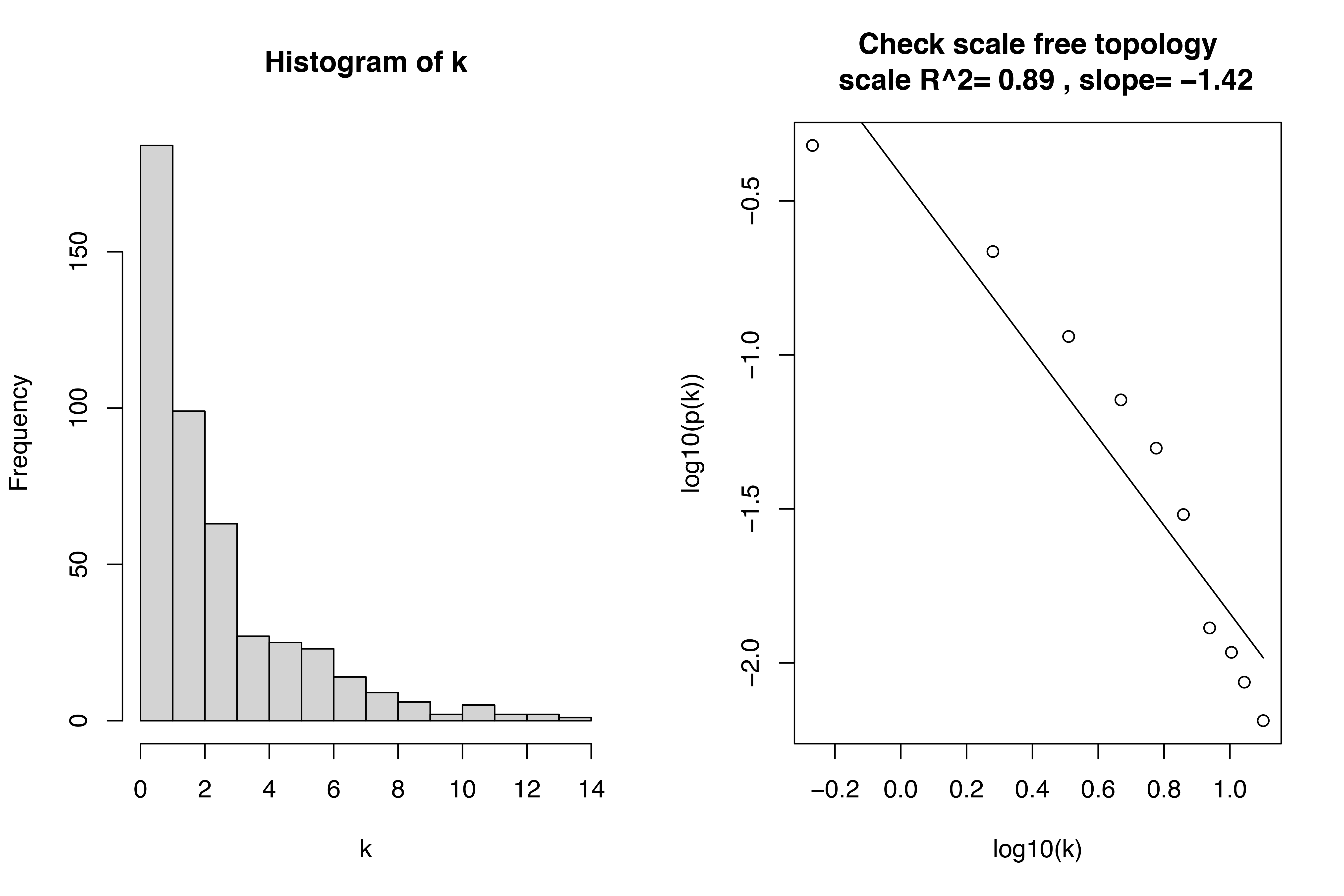

Supplement: Supplementary file 1 [file Image2.TIF]

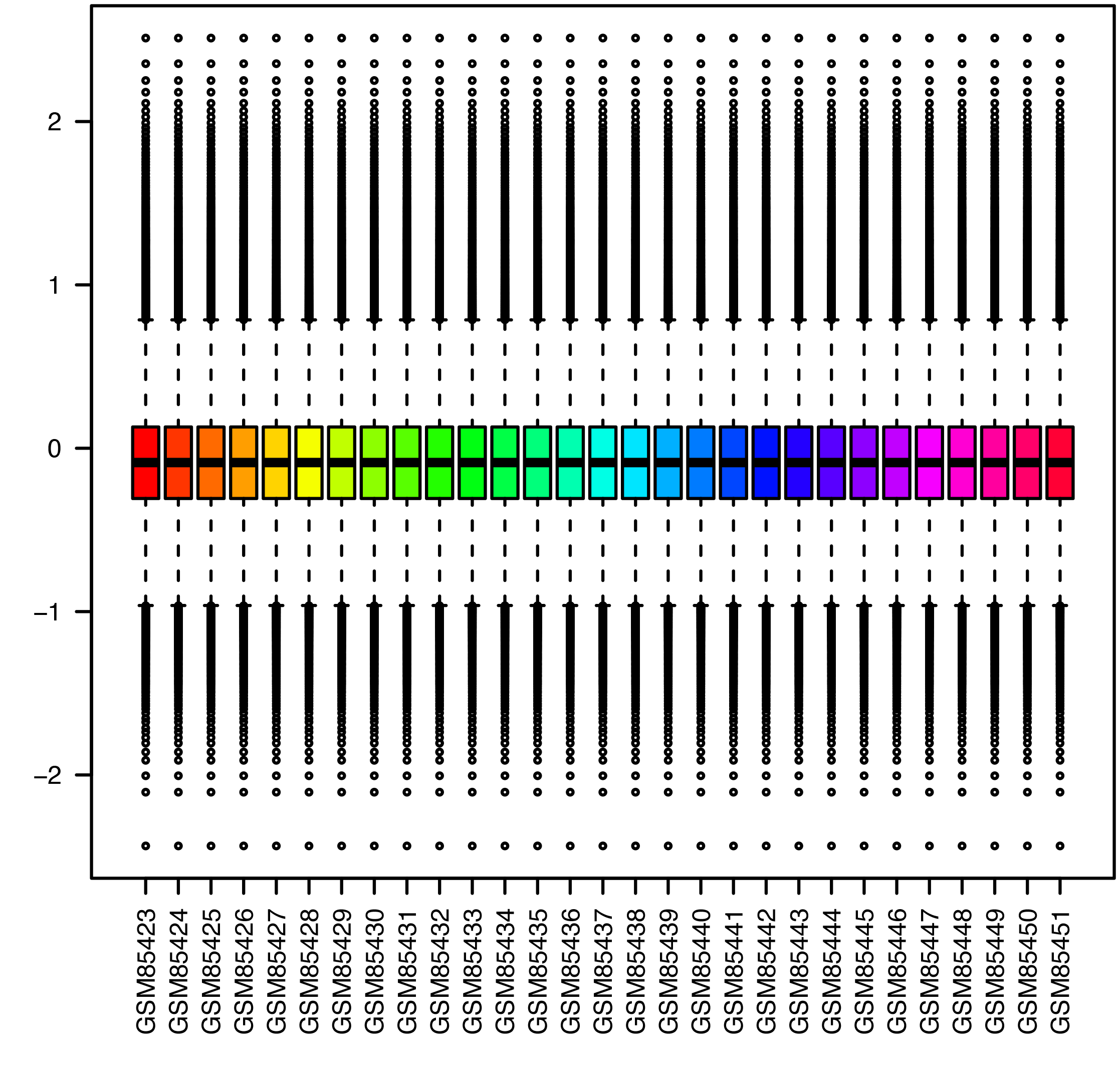

Supplement: Supplementary file 2 [file Image1.TIF]
